# Antifungal Activity of Salicylanilides and Their Esters with 4-(Trifluoromethyl)benzoic Acid

**DOI:** 10.3390/molecules17089426

**Published:** 2012-08-07

**Authors:** Martin Krátký, Jarmila Vinšová

**Affiliations:** Department of Inorganic and Organic Chemistry, Faculty of Pharmacy, Charles University, Heyrovského 1203, Hradec Králové 50005, Czech Republic; Email: martin.kratky@faf.cuni.cz

**Keywords:** antifungal activity, *in vitro* activity, salicylanilide, salicylanilide ester, 4-(trifluoromethyl)benzoic acid ester

## Abstract

Searching for novel antimicrobial agents still represents a current topic in medicinal chemistry. In this study, the synthesis and analytical data of eighteen salicylanilide esters with 4-(trifluoromethyl)benzoic acid are presented. They were assayed *in vitro* as potential antimycotic agents against eight fungal strains, along with their parent salicylanilides. The antifungal activity of the presented derivatives was not uniform and moulds showed a higher susceptibility with minimum inhibitory concentrations (MIC) ≥ 0.49 µmol/L than yeasts (MIC ≥ 1.95 µmol/L). However, it was not possible to evaluate a range of 4-(trifluoromethyl)benzoates due to their low solubility. In general, the most active salicylanilide was *N*-(4-bromophenyl)-4-chloro-2-hydroxybenzamide and among esters, the corresponding 2-(4-bromophenylcarbamoyl)-5-chlorophenyl 4-(trifluoromethyl) benzoate exhibited the lowest MIC of 0.49 µmol/L. However, the esterification of salicylanilides by 4-(trifluoromethyl)benzoic acid did not result unequivocally in a higher antifungal potency.

## 1. Introduction

The spread of fungal infections has recently been increasing because of the eminent presence of the predisposing risk factors, e.g., malnutrition, invasive surgical procedures, treatment with broad-spectrum antibiotics, corticosteroids or other immunosuppressive agents, immunodeficiency related to diseases like diabetes mellitus or human immunodeficiency virus infections. Mycoses may either be superficial or systemic [1,2]. Immunocompromised patients can develop opportunistic mycoses caused by an expanding spectrum of fungal pathogens, including those with problematic susceptibility to current antifungal drugs [3]. Invasive fungal infections are a major cause of morbidity and mortality in these patients. *Candida albicans*, *Cryptococcus neoformans* and *Aspergillus fumigatus* comprise the most common etiologic agents, but there is an increasing number of infections caused by rare pathogens, including e.g., non-*albicans Candida* species, opportunistic yeast-like fungi like *Trichosporon* spp. or non-*fumigatus Aspergillus* spp. [4].

Pathogenic fungi can also develop resistance to various drugs with unshared mechanisms of action [3]. That is why the development of new antifungal agents still remains challenging, despite the intensive searching in recent times.

Salicylanilides (2-hydroxy-*N*-phenylbenzamides) have displayed a wide range of potentially interesting biological effects including those against protozoa, fungi, bacteria and mycobacteria or viruses [5,6]. Their mechanism of the action towards microbes is believed to be multiple, with a lot of molecular targets and effects [7].

Waisser *et al*. [8] reported the activity of twelve salicylanilides with various substitution patterns towards nine fungal strains. Minimum inhibitory concentrations (MIC) for yeast strains were proven to be ≥15.63 µmol/L and ≥3.91 µmol/L for filamentous fungi, respectively; however, some derivatives only exhibited low activity. The most susceptible strains were determined to be *Trichophyton mentagrophytes* and *Microsporum gypseum*. Other authors have also reported rather moderate or mild antifungal activity of salicylanilides, e.g., towards *Absidia corymbifera* and *Trichophyton mentagrophytes* [9], *Sacharomyces cerevisiae* and *C. albicans* (MIC of 0.3125–10 mmol/L) [10], or from another source, 2–3.2 µmol/L [11], or 25 μg/mL against *C. albicans* [12]. *Aspergillus sp.* was also partly susceptible [13]. 2,3-Dihydroxy-*N*-(substituted)phenylbenzamides and 2,6-dihydroxy-*N*-(substituted)phenylbenzamide derivatives (“dihydroxysalicylanilides”) also showed a significant antifungal potency with MIC values ranging between 3.91–250 µmol/L for *Candida* species and 1.95–250 µmol/L for filamentous fungi [14].

The temporary masking by an esterification of a salicylanilide free phenolic group, which is presumed to be necessary for the antimicrobial activity, may be beneficial, e.g., due to improved bioavailability and membrane permeability, a high activity and/or a lower toxicity [7]. A correlation between hydrophilic/lipophilic balance (lipophilicity) necessary to penetrate through biological barriers and the antimicrobial activity with an optimal span has been described repeatedly [15,16].

The antifungal properties of salicylanilide esters (acetates, esters with *N*-protected amino acids) were summarized in our earlier review [7]; later, a mild and sporadic activity was found for salicylanilide benzoates (MIC ≥ 3.9 µmol/L) [17] and more importantly for salicylanilide pyrazinoates (MIC ≥ 1.95 µmol/L) [18] with a general result that salicylanilide esters are more active and probably fungicidal against filamentous fungi than against *Candida* strains with only a limited fungistatic activity.

Based on these facts, we synthesised a new series of salicylanilide esters with 4-(trifluoro-methyl) benzoic acid (illustratively, esters of imidazole derivatives with 3- and 4-(trifluoro-methyl)benzoic acids expressed some activity against *Candida* strains [19]) and evaluated them as well as their parent salicylanilides as potential antifungal agents.

## 2. Results and Discussion

### 2.1. Chemistry

**Scheme 1:**
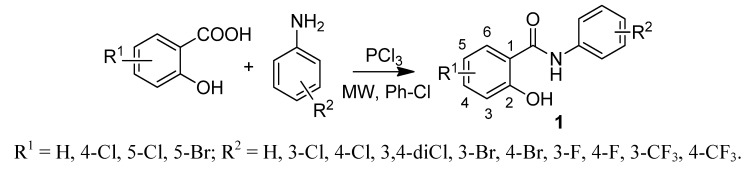
Synthesis of salicylanilides.

**Scheme 2:**
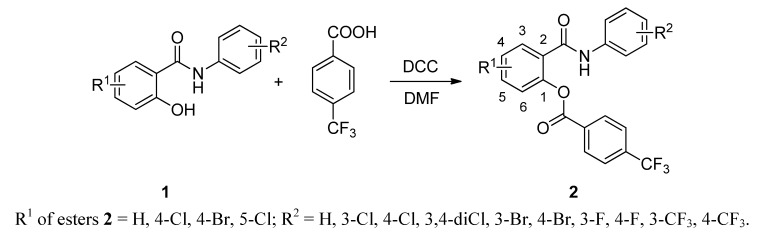
Synthesis of salicylanilide 4-(trifluoromethyl)benzoates.

Salicylanilides and their esters with 4-(trifluoromethyl)benzoic acid were obtained by two procedures used previously by our group [5,20]. Firstly, salicylanilides **1** were prepared by the reaction of appropriate salicylic acids and anilines in the presence of PCl_3_ in chlorobenzene (Ph-Cl) under microwave irradiation (Scheme 1). Then they were esterified by 4-(trifluoromethyl)benzoic acid via *N*,*N*′-dicyclohexylcarbodiimide (DCC) coupling in dry *N*,*N*-dimethylformamide (DMF), see Scheme 2. Yields of salicylanilides ranged within 75–90%, of esters within 49–86%.

### 2.2. *In vitro* Antifungal Evaluation

*In vitro* antifungal properties of thirty seven salicylanilide derivatives were assayed against four *Candida* strains (*Candida albicans* ATCC 44859, *Candida tropicalis* 156, *Candida krusei* E28, and *Candida glabrata* 20/I), *Trichosporon asahii* 1188 and three filamentous fungi (*Aspergillus fumigatus* 231, *Absidia corymbifera* 272, and *Trichophyton mentagrophytes* 445).

Although it was our intention, the sharply increased lipophilicity of salicylanilide 4-(trifluoro-methyl) benzoates (e.g., Clog*P* of 4.49 for **1n** *vs.* 6.02 for **2n**) caused problems with the biological assessment. In contrast to the parent salicylanilides, some of the esters (ten derivatives: **2a**, **2b**, **2d**, **2g**, **2i**, **2l**, **2m**, **2n**, **2o**, **2s**) did not dissolve very well in the testing medium or they precipitated after a very short period; therefore it was not possible to assay their MIC values. Table 1 and Table 2 overview the antifungal properties, and Table 1 for yeasts, Table 2 for filamentous fungi. 4-(Trifluoro-methyl)benzoic acid itself was completely inactive for all species under our conditions.

**Table 1:** *In vitro* activity of salicylanilides **1** and their 4-(trifluoromethyl)benzoates **2** towards yeasts.

MIC/IC *80* [μmol/L]													
				*Candida albicans*		*Candida tropicalis*		*Candida krusei*		*Candida glabrata*		*Trichosporon asahii*	
Code	R^1^	R^2^	Clog *P*	24 h	48 h	24 h	48 h	24 h	48 h	24 h	48 h	24 h	48 h
**1a**	5-Cl	3-Cl	5.85	>125	>125	>125	>125	>125	>125	>125	>125	>125	>125
**2a**	4-Cl	3-Cl	7.02	ND	ND	ND	ND	ND	ND	ND	ND	ND	ND
**1b**	4-Cl	3-Cl	5.18	>125	>125	>125	>125	>125	>125	>125	>125	>125	>125
**2b**	5-Cl	3-Cl	6.71	ND	ND	ND	ND	ND	ND	ND	ND	ND	ND
**1c**	5-Cl	4-Cl	5.50	>125	>125	>125	>125	>125	>125	>125	>125	>125	>125
**2c**	4-Cl	4-Cl	6.68	62.5	>250	>250	>250	250	>250	>250	>250	250	>250
**1d**	4-Cl	4-Cl	4.83	>125	>125	>125	>125	>125	>125	>125	>125	>125	>125
**2d**	5-Cl	4-Cl	6.37	ND	ND	ND	ND	ND	ND	ND	ND	ND	ND
**1e**	5-Cl	3,4-diCl	6.51	>125	>125	>125	>125	>125	>125	>125	>125	>125	>125
**2e**	4-Cl	3,4-diCl	7.69	>500	>500	**7.81**	**31.25**	**7.81**	**31.25**	>500	>500	>500	>500
**1f**	4-Cl	3,4-diCl	5.84	>125	>125	>125	>125	>125	>125	>125	>125	**3.9**	15.62
**2f**	5-Cl	3,4-diCl	7.38	>125	>125	>125	>125	62.5	>125	>125	>125	31.25	>125
**1g**	5-Cl	3-Br	5.92	>125	>125	>125	>125	>125	>125	>125	>125	15.62	62.5
**2g**	4-Cl	3-Br	7.09	ND	ND	ND	ND	ND	ND	ND	ND	ND	ND
**1h**	4-Cl	3-Br	5.25	>125	>125	>125	>125	>125	>125	>125	>125	**1.95**	62.5
**2h**	5-Cl	3-Br	6.87	125	>500	>500	>500	125	250	>500	>500	62.5	250
**1i**	5-Cl	4-Br	5.88	>500	>500	>500	>500	>500	>500	>500	>500	**3.9**	**3.9**
**2i**	4-Cl	4-Br	7.05	ND	ND	ND	ND	ND	ND	ND	ND	ND	ND
**1j**	4-Cl	4-Br	5.21	**7.81**	>125	>125	>125	**7.81**	>125	>125	>125	**1.95**	7.81
**2j**	5-Cl	4-Br	6.74	**15.62**	31.25	500	>500	**31.25**	62.5	500	>500	15.62	31.25
**1k**	5-Cl	3-F	5.20	>125	>125	>125	>125	>125	>125	>125	>125	>125	>125
**2k**	4-Cl	3-F	6.37	250	>250	>250	>250	>250	>250	>250	>250	>250	>250
**1l**	4-Cl	3-F	4.53	>125	>125	>125	>125	>125	>125	>125	>125	>125	>125
**2l**	5-Cl	3-F	6.06	ND	ND	ND	ND	ND	ND	ND	ND	ND	ND
**1m**	5-Cl	4-F	5.16	>125	>125	>125	>125	>125	>125	>125	>125	>125	>125
**2m**	4-Cl	4-F	6.33	ND	ND	ND	ND	ND	ND	ND	ND	ND	ND
**1n**	4-Cl	4-F	4.49	>125	>125	>125	>125	>125	>125	>125	>125	>125	>125
**2n**	5-Cl	4-F	6.02	ND	ND	ND	ND	ND	ND	ND	ND	ND	ND
**1o**	5-Cl	3-CF_3_	6.06	>125	>125	>125	>125	>125	>125	>125	>125	>125	>125
**2o**	4-Cl	3-CF_3_	7.24	ND	ND	ND	ND	ND	ND	ND	ND	ND	ND
**1p**	4-Cl	3-CF_3_	5.39	>500	>500	>500	>500	>500	>500	500	>500	250	500
**2p**	5-Cl	3-CF_3_	6.93	>500	>500	>500	>500	>500	>500	>500	>500	>500	>500
**1q**	5-Cl	4-CF_3_	5.72	>125	>125	>125	>125	>125	>125	>125	>125	>125	>125
**2q**	4-Cl	4-CF_3_	6.90	>125	>125	>125	>125	>125	>125	>125	>125	>125	>125
**1r**	4-Cl	4-CF_3_	5.05	>125	>125	>125	>125	>125	>125	>125	>125	**1.95**	15.62
**2r**	5-Cl	4-CF_3_	6.59	62.5	500	>500	>500	**31.25**	62.5	>500	>500	15.62	62.5
**1s**	5-Br	4-CF_3_	5.49	>125	>125	>125	>125	>125	>125	>125	>125	**1.95**	7.81
**2s**	4-Br	4-CF_3_	6.50	ND	ND	ND	ND	ND	ND	ND	ND	ND	ND
**1t**	H	H	3.27	250	500	250	500	250	500	250	250	250	500
**UND**				>500	>500	>500	>500	>500	>500	>500	>500	250	>500
**FLU**				1.0	2.0	3.0	5.0	>50	>50	22.0	>50	4.00	9.00

The best MIC values are highlighted. UND: undecylenic acid; FLU: fluconazole.

**Table 2:** *In vitro* activity of salicylanilides **1** and their 4-(trifluoromethyl)benzoates **2** towards moulds.

MIC/IC_80_/IC_50_ [μmol/L]								
			*Aspergillus fumigatus*		*Absidia corymbifera*		*Trichophyton mentagrophytes*	
Code	R^1^	R^2^	24 h	48 h	24 h	48 h	72 h	150 h
**1a**	5-Cl	3-Cl	>125	>125	**7.81**	62.5	1.95	1.95
**2a**	4-Cl	3-Cl	ND	ND	ND	ND	ND	ND
**1b**	4-Cl	3-Cl	>125	>125	125	>125	1.95	1.95
**2b**	5-Cl	3-Cl	ND	ND	ND	ND	ND	ND
**1c**	5-Cl	4-Cl	>125	>125	125	>125	1.95	1.95
**2c**	4-Cl	4-Cl	>250	>250	250	250	15.62	15.62
**1d**	4-Cl	4-Cl	>125	>125	15.62	>125	1.95	1.95
**2d**	5-Cl	4-Cl	ND	ND	ND	ND	ND	ND
**1e**	5-Cl	3,4-diCl	>125	>125	>125	>125	31.25	31.25
**2e**	4-Cl	3,4-diCl	>500	>500	>500	>500	15.62	125
**1f**	4-Cl	3,4-diCl	>125	>125	**3.9**	**7.81**	3.9	3.9
**2f**	5-Cl	3,4-diCl	>125	>125	>125	>125	>125	>125
**1g**	5-Cl	3-Br	>125	>125	15.62	31.25	1.95	1.95
**2g**	4-Cl	3-Br	ND	ND	ND	ND	ND	ND
**1h**	4-Cl	3-Br	>125	>125	>125	>125	1.95	1.95
**2h**	5-Cl	3-Br	>500	>500	250	>500	**0.98**	1.95
**1i**	5-Cl	4-Br	>500	>500	15.62	31.25	**0.98**	**0.98**
**2i**	4-Cl	4-Br	ND	ND	ND	ND	ND	ND
**1j**	4-Cl	4-Br	>125	>125	**7.81**	**7.81**	1.95	1.95
**2j**	5-Cl	4-Br	**15.62**	**31.25**	**7.81**	15.62	**0.49**	**0.98**
**1k**	5-Cl	3-F	>125	>125	>125	>125	3.9	3.9
**2k**	4-Cl	3-F	>250	>250	250	>250	62.5	62.5
**1l**	4-Cl	3-F	>125	>125	>125	>125	3.9	3.9
**2l**	5-Cl	3-F	ND	ND	ND	ND	ND	ND
**1m**	5-Cl	4-F	>125	>125	>125	>125	7.81	7.81
**2m**	4-Cl	4-F	ND	ND	ND	ND	ND	ND
**1n**	4-Cl	4-F	>125	>125	>125	>125	62.5	62.5
**2n**	5-Cl	4-F	ND	ND	ND	ND	ND	ND
**1o**	5-Cl	3-CF3	>125	>125	**3.9**	**3.9**	3.9	3.9
**2o**	4-Cl	3-CF3	ND	ND	ND	ND	ND	ND
**1p**	4-Cl	3-CF3	>500	>500	>500	>500	>500	>500
**2p**	5-Cl	3-CF3	>500	>500	>500	>500	15.62	31.25
**1q**	5-Cl	4-CF3	>125	>125	>125	>125	3.9	7.81
**2q**	4-Cl	4-CF3	>125	>125	>125	>125	3.9	15.62
**1r**	4-Cl	4-CF3	>125	>125	**3.9**	**3.9**	3.9	3.9
**2r**	5-Cl	4-CF3	**15.62**	**15.62**	15.62	15.62	**0.49**	1.95
**1s**	5-Br	4-CF3	>125	>125	**3.9**	**7.81**	7.81	7.81
**2s**	4-Br	4-CF3	ND	ND	ND	ND	ND	ND
**1t**	H	H	500	500	250	500	31.25	125
**UND**			>500	>500	>500	>500	250	250
**FLU**			>50	>50	>50	>50	17.0	26.0

The best MIC values are highlighted. UND: undecylenic acid; FLU: fluconazole.

None of the compounds in this study inhibited *Candida glabrata* at 125 µmol/L, while for other strains some activity was found. Four esters (**2c**, **2h**, **2j**, **2r**) and two salicylanilides (**1j**, **1t**) affected *Candida albicans* up to the concentration of 500 µmol/L with **1j** (7.81 µmol/L) and **2j** showing superiority. Only ester **2e** exhibited a significant activity towards *C. tropicalis* at low concentrations (7.81/31.25 µmol/L). *C. crusei* was inhibited by seven molecules (**2e**, **2f**, **2h**, **1j**, **2j**, **2r**, **1t**) from 7.81 µmol/L. Fourteen salicylanilide derivatives inhibited *Trichosporon asahii*, the most susceptible yeast with MIC values ranging between 1.95–500 µmol/L. None of the presented derivatives reached the activity of fluconazole against *C. albicans*, *C. tropicalis* and *C. glabrata*, whereas four showed for *C. krusei* a lower MIC than fluconazole. Additionally, four derivatives surpassed the MIC of fluconazole for *T. asahii* after 24 h of incubation (**1h**, **1j**, **1r**, **1s**; **1f** and **1i** were comparable) and three salicylanilides after 48 h (**1i**, **1j**, **1s**). Undecylenic acid, an established non-specific antimycotic agent for topical usage [21], exhibited mostly MIC values ≥ 500 µmol/L (except 250 µmol/L for *T. asahii*).

Based on earlier findings [5,16,18], we expected a higher activity of the salicylanilide scaffold-based molecules towards moulds. However, only two esters (**2j**, **2r**) inhibited the growth of *A. fumigatus* at 15.62–31.25 µmol/L. Seventeen compounds affected *A. corymbifera* with MICs of 3.9–500 µmol/L (salicylanilides **1a**, **1b**, **1c**, **1d**, **1f**, **1g**, **1i**, **1j**, **1o**, **1r**, **1s**, **1t** and five esters **2c**, **2h**, **2j**, **2k**, **2r**); only one ester **2f** and one salicylanilide **1p** did not exhibit any activity towards *Trichophyton mentagrophytes*. This strain was assayed as being the most susceptible (MIC ≥ 0.49 µmol/L) from the evaluated panel of fungi. Moreover, based on a comparison of MIC values, the mechanism of the action seems to be fungicidal like against *A. corymbifera* and in contrast to *Candida* strains. Twenty derivatives (**1a**, **1b**, **1c**, **1d**, **1f**, **1g**, **1h**, **2h**, **1i**, **1j**, **2j**, **1k**, **1l**, **1m**, **1o**, **1q**, **2q**, **1r**, **2r**, **1s**) surpassed the activity of fluconazole towards *T. mentagrophytes* with **1i** and **2j** showing superiority (0.49–0.98 µmol/L). Undecylenic acid stopped the growth of *T. mentagrophytes* at 250 µmol/L.

In general, the most antifungal active salicylanilide assayed was *N*-(4-bromophenyl)-4-chloro-2-hydroxybenzamide (**1j**). Bromine and 4-trifluoromethyl moiety-containing salicylanilides expressed mostly a significant activity towards *T. asahii*, *A. corymbifera* and *T. mentagrophytes*, while dichlorosalicylanilides were only active against the last strain. It is difficult to postulate distinct structure-activity relationships due to a lot of partially inactive derivatives; for some strains the activity is only sporadic.

Similarly, the fact that for most of the esters it was not possible to determine their MIC values, complicates the systematic evaluation of how the esterification by 4-(trifluoromethyl)benzoic acid influenced the activity of the parent salicylanilides. In some cases the esterification improved the activity (e.g., **1e** *vs.* **2e** and **1r** *vs.* **2r** for *Candida* sp., **1j** *vs.* **2j** for filamentous fungi, **1p** *vs.* **2p** for *T. mentagrophytes*), for others the impact was the reverse (e.g., **1j** *vs.* **2j** for yeasts, **1c** *vs.* **2c** for moulds, **1f** *vs.* **2f**, **1k** *vs.* **2k**). However, only the esters inhibited *A. fumigatus*, *C. tropicalis* and also *C. krusei* (additionally with **1j**) at low MIC values. Additionally, some 4-(trifluoromethyl)benzoates (**2h**, **2j**, **2r**) share submicromolar MICs for *T. mentagrophytes* after 72 h of incubation.

Although lipophilicity is generally presumed to belong to the factors influencing antifungal activity, there is no sharp correlation in this series, as demonstrated above for the pairs of salicylanilide *vs.* its ester. The most lipophilic salicylanilide **1e** was not the unambiguously most active derivative; its MIC values were even below-average. Similarly, the least lipophilic fluorinated salicylanilides **1k**, **1l**, **1m** and **1n** showed a better activity in some cases than more lipophilic molecules like **1e** or **1p**. Similarly, compounds with a comparable calculated log*P* showed non-uniform MIC values (e.g., **1b** *vs.* **1j**, **2h** *vs.* **2p**). Moreover, if the antifungal properties depend only on the lipophilicity, the salicylanilides derived from 5-chlorosalicylic acid and 3-substituted anilines should be unanimously more active than those synthesized from 4-chlorosalicylic acid and 4-substituted anilines; but this clear relationship was not observed and *N*-(4-bromophenyl)-4-chloro-2-hydroxybenzamide (**1j**) revealed the most significant *in vitro* antifungal features. However, the log*P* values presented here are only calculated values, not experimentally found; therefore their significance should be viewed with caution.

Similarly, it was shown that the individual biological impacts of related compounds with various substitution patterns may be in part often influenced by the volume of the substituents. This bulkiness can be expressed, e.g., by a bulk parameter like MR (molar refractivity) [9,22]. The esterification of salicylanilides by 4-(trifluoromethyl)benzoic acid led to the larger molecules with a calculated increment of MR of 34.74. However, as pointed out previously, this modification did not improve the activity unequivocally. Similarly, bulkier derivatives **1e**, **1f**, **2e** and **2f** with two chlorines on the aniline ring (MR for these atoms together = 9.6) did not present uniformly superior activity, and only some MICs are excellent, when the remainder are average, weak or comparatively high. On the other hand, the replacement of chlorine (MR = 4.8) by a bulkier bromine (MR = 7.6) on the salicylic acid ring resulted in the improvement of the activity towards *T. asahii* and *A. corymbifera* (**1q** *vs.* **1s**), while for other strains this brought no additional benefit. Similarly, anilines containing bromine (MR = 7.6) showed a higher antifungal activity than those substituted by chlorine (4.8), fluorine (−0.4) and it is slightly superior to a trifluoromethyl group (MR = 4.0). These data indicate, analogously to lipophilicity, that there is no simple linear correlation between biological activity and steric parameter MR.

When compared to other recently evaluated salicylanilide esters, 4-(trifluoromethyl)benzoates did not surpass the activity of salicylanilide acetates [16], although some MICs are comparable. Contrarily, benzoates [17] are much less potent *in vitro* than the here presented esters. The comparison with salicylanilide pyrazinoates [18] and esters with *N*-benzyloxycarbonyl amino acids [23] is not unambiguous, while these derivatives showed more consistent activity; however, MICs of many 4-(trifluoromethyl)benzoates exceeded them. Esters with *N*-acetyl-L-phenylalanine showed less and less uniform antifungal potency [5]. In some cases, the esterification of parent salicylanilides by various carboxylic acids led to a decreased activity.

## 3. Experimental

### 3.1. General Methods

All the reagents and solvents were purchased from Sigma-Aldrich (Seelze, Germany) or Penta Chemicals (Prague, Czech Republic) and they were used as received. Reactions and purity of products were monitored by thin layer chromatography with a toluene/ethyl-acetate 4:1 mixture as eluent for salicylanilides and a toluene/methanol 9:1 mixture as eluent for esters; plates were coated with 0.2 mm Merck 60 F254 silica gel and were visualized by UV irradiation (254 nm). Melting points were determined on a Büchi Melting Point machine B-540 apparatus using open capillaries and the reported values are uncorrected.

Elemental analysis (C, H, N) were performed on an automatic microanalyser CHNS-O CE instrument (FISONS EA 1110, Milano, Italy). Infrared spectra (ATR) were recorded on FT-IR spectrometer Nicolet 6700 FT-IR in the range of 400–4,000 cm^−1^. The NMR spectra were recorded on a Varian Mercury-Vxbb 300 (300 MHz for ^1^H and 75.5 MHz for ^13^C; Varian, Inc., Palo Alto, CA, USA) at ambient temperature using deuterated dimethylsulfoxide (DMSO-*d_6_*) solutions of the samples. The chemical shifts δ are given in ppm, with respect to tetramethylsilane as an internal standard. The coupling constants (*J*) are reported in Hz.

The calculated log*P* values (Clog*P*), that are the logarithms of the partition coefficients for octan-1-ol/water, were determined using the program ACD/ChemSketch (Freeware) version 12.01 (Advanced Chemistry Development, Inc., Toronto, ON, Canada); while the program CS ChemOffice Ultra version 12.0 (CambridgeSoft, Cambidge, MA, USA) did not allow us to distinguish lipophilicity for particular position isomers providing the same log*P* data. Only the mean values without deviations are reported in this work. The increment of molar refractivity due to esterification of salicylanilides by 4-(trifluoromethyl)benzoic acid was calculated using the program ACD/ChemSketch (Freeware) version 12.01.

### 3.2. Synthesis of Salicylanilides

A substituted salicylic acid and corresponding aniline (both 0.002 mol) were suspended in chlorobenzene (20 mL) and PCl_3_ (0.001 mol) was added. The reaction was carried out with vigorously stirring in a microwave reactor (530 W, 600 rpm, MicroSYNTH Milestone) for 20 min to refluxing. The reaction mixture was filtered while hot, let stand at 20 °C and then at +4 °C for 24 h. The crude product was filtered off and recrystallized from boiling 96% ethanol to obtain the pure product, in all cases white crystals. Salicylanilides, whose physical and spectral properties are not presented here, were synthesized and reported previously [24,25].

*N-(3-Bromophenyl)-4-chloro-2-hydroxybenzamide* (**1h**). Yield 79%; m.p.: 224.5–226.5 °C. IR: 3307, 1608 (CO amide), 1547, 1497, 1425, 1376, 1228, 1204, 1093, 1070, 927, 861, 848, 777, 762, 717, 677. ^1^H-NMR: δ 11.87 (1H, s, OH), 10.42 (1H, bs, NH), 8.04 (1H, s, H2′), 7.88 (1H, d, *J* = 8.3 Hz, H6), 7.67–7.60 (1H, m, H6′), 7.32–7.29 (2H, m, H4′, H5′), 7.05–7.00 (2H, m, H3, H5). ^13^C-NMR: δ 165.6, 158.7, 140.0, 137.6, 131.2, 130.9, 126.9, 123.1, 121.7, 119.6, 119.5, 117.8, 116.9. Anal. Calcd. for C_13_H_9_BrClNO_2_ (326.57): C, 47.81; H, 2.78; N, 4.29. Found: C, 47.99; H, 2.83; N, 4.10.

*4-Chloro-2-hydroxy-N-[3-(trifluoromethyl)phenyl]benzamide* (**1p**). Yield 85%; m.p.: 193–195 °C. IR: 3322, 3057, 1633, 1614 (CO amide), 1603, 1656, 1494, 1423, 1334, 1313, 1223, 1170, 1124, 1099, 1072, 923, 891, 864, 794, 711, 698. ^1^H-NMR: δ 11.83 (1H, s, OH), 10.56 (1H, bs, NH), 8.20 (1H, s, H2′), 7.94–7.87 (2H, m, H6, H6′), 7.59 (1H, t, *J* = 8.0 Hz, H5′), 7.46 (1H, d, *J* = 7.8 Hz, H4′), 7.05–7.01 (2H, m, H3, H5). ^13^C-NMR: δ 165.9, 158.8, 139.2, 137.7, 131.2, 130.1, 129.7 (q, *J* = 31.8 Hz), 124.4, 124.3 (q, *J* = 272.6 Hz), 120.6 (q, *J* = 3.8 Hz), 119.5, 117.8, 116.9 (q, *J* = 3.8 Hz). Anal. Calcd. for C_14_H_9_ClF_3_NO_2_ (315.67): C, 53.27; H, 2.87; N, 4.44. Found: C, 53.55; H, 2.70; N, 4.55.

### 3.3. Synthesis of Salicylanilide 4-(Trifluoromethyl)benzoates

4-(Trifluoromethyl)benzoic acid and appropriate salicylanilide (both 0.001 mol) were dissolved in dry DMF (15 mL), the solution was cooled to −20 °C and DCC in a mild excess (0.0012 mol) was added in three portions during 1 h. Next the mixture was stirred for 3 h at the same temperature and stored at +4 °C for 48 h. After this time, the precipitated by-product *N*,*N*′-dicyclohexylurea was filtered off and the solvent was evaporated *in vacuo*. The rest was dissolved in a small amount of ethyl-acetate and the insoluble portion (another *N*,*N*′-dicyclohexylurea) was filtered off. The filtrate was again evaporated *in vacuo*. The crude product was purified by the repeated crystallization from ethyl-acetate/hexane; all esters are white solids.

*4-Chloro-2-(3-chlorophenylcarbamoyl)phenyl-4-(trifluoromethyl)benzoate* (**2a**). Yield 63%; m.p.: 184–186 °C. IR (ATR): 3308, 2934, 2858, 1718 (CO ester), 1666, 1650, 1588, 1522, 1479, 1452, 1425, 1410, 1323, 1291, 1271, 1205, 1167, 1131, 1113, 1088, 1066, 1015, 861, 766, 693, 680. ^1^H-NMR: δ 10.71 (1H, bs, NH), 8.26 (2H, d, *J* = 8.1 Hz, H2′′, H6′′), 7.94 (2H, d, *J* = 8.3 Hz, H3′′, H5′′), 7.87 (1H, d, *J* = 2.6 Hz, H3), 7.77–7.71 (3H, m, H5, H6, H2′), 7.54 (1H, d, *J* = 8.7 Hz, H6′), 7.30 (1H, t, *J* = 8.1 Hz, H5′), 7.11 (1H, ddd, *J* = 0.9 Hz, *J* = 2.1 Hz, *J* = 8.0 Hz, H4′). ^13^C-NMR: δ 163.3, 162.9, 146.9, 140.2, 133.7 (q, *J* = 32.1 Hz), 133.1, 132.4, 131.9, 130.9, 130.8, 130.7, 130.6, 129.3, 126.2 (q, *J* = 3.7 Hz), 125.6, 123.8, 123.7 (q, *J* = 272.9 Hz), 119.5, 118.4. Anal. Calcd. for C_21_H_12_Cl_2_F_3_NO_3_ (454.23): C, 55.53; H, 2.66; N, 3.08. Found: C, 55.27; H, 2.80; N, 3.22.

*5-Chloro-2-(3-chlorophenylcarbamoyl)phenyl-4-(trifluoromethyl)benzoate* (**2b**). Yield 83%; m.p.: 165.5–168 °C. IR (ATR): 3284, 2933, 2857, 1738 (CO ester), 1650, 1602, 1593, 1543, 1483, 1451, 1413, 1323, 1259, 1242, 1168, 1130, 1112, 1068, 1016, 860, 699, 673. ^1^H-NMR: δ 10.67 (1H, bs, NH), 8.27 (2H, d, *J* = 8.1 Hz, H2′′, H6′′), 7.94 (2H, d, *J* = 8.3 Hz, H3′′, H5′′), 7.82 (1H, d, *J* = 8.3 Hz, H3), 7.73–7.71 (2H, m, H6, H2′), 7.60 (1H, dd, *J* = 8.3 Hz, *J* = 2.1 Hz, H4), 7.50 (1H, dd, *J* = 8.3 Hz, *J* = 2.0, H6′), 7.30 (1H, t, *J* = 8.1 Hz, H5′), 7.11 (1H, m, H4′). ^13^C-NMR: δ 163.4, 163.2, 148.8, 140.3, 136.0, 133.8 (q, *J* = 32.1 Hz), 133.1, 132.4, 131.1, 130.9, 130.6, 128.3, 126.7, 126.2 (q, *J* = 3.6 Hz), 124.0, 123.9 (q, *J* = 272.9 Hz), 123.8, 119.4, 118.4. Anal. Calcd. for C_21_H_12_Cl_2_F_3_NO_3_ (454.23): C, 55.53; H, 2.66; N, 3.08. Found: C, 55.34; H, 2.85; N, 3.01.

*4-Chloro-2-(4-chlorophenylcarbamoyl)phenyl-4-(trifluoromethyl)benzoate* (**2c**). Yield 49%; m.p.: 170–172.5 °C. IR (ATR): 3322, 3073, 2934, 1719 (CO ester), 1667, 1625, 1601, 1547, 1491, 1420, 1399, 1324, 1291, 1244, 1221, 1172, 1134, 1112, 1095, 1067, 1013, 822, 705, 656. ^1^H-NMR: δ 10.67 (1H, bs, NH), 8.25 (2H, d, *J* = 8.2 Hz, H2′′, H6′′), 7.93 (2H, d, *J* = 8.3 Hz, H3′′, H5′′), 7.86 (1H, d, *J* = 2.6 Hz, H3), 7.73 (1H, dd, *J* = 8.8 Hz, *J* = 2.1 Hz, H5), 7.62 (1H, d, *J* = 8.9 Hz, H6), 7.54–7.40 (2H, m, H2′, H6′), 7.34–7.31 (2H, m, H3′, H5′). ^13^C-NMR: δ 163.3, 162.7, 146.9, 137.8, 133.7 (q, *J* = 32.0 Hz), 133.2, 130.9, 130.8, 130.3, 129.2, 128.8, 128.0, 126.2 (q, *J* = 3.6 Hz), 125.6, 123.9 (q, *J* = 272.8 Hz), 121.6. Anal. Calcd. for C_21_H_12_Cl_2_F_3_NO_3_ (454.23): C, 55.53; H, 2.66; N, 3.08. Found: C, 55.40; H, 2.51; N, 3.34.

*5-Chloro-2-(4-chlorophenylcarbamoyl)phenyl-4-(trifluoromethyl)benzoate* (**2d**). Yield 86%; m.p.: 170.5–173 °C. IR (ATR): 3305, 2933, 2858, 1737 (CO ester), 1699, 1650, 1601, 1544, 1492, 1452, 1408, 1325, 1258, 1240, 1182, 1157, 1128, 1111, 1097, 1068, 1016, 829, 769, 698, 669. ^1^H-NMR: δ 10.63 (1H, bs, NH), 8.26 (2H, d, *J* = 8.1 Hz, H2′′, H6′′), 7.95 (2H, d, *J* = 8.3 Hz, H3′′, H5′′), 7.82 (1H, d, *J* = 8.3 Hz, H3), 7.72 (1H, d, *J* = 2.0 Hz, H6), 7.54–7.40 (3H, m, H4′, H2′, H6′), 7.34–7.30 (2H, m, H3′, H5′). ^13^C-NMR: δ 163.6, 163.2, 148.9, 136.1, 133.7 (q, *J* = 32.0 Hz), 132.4, 131.1, 130.8, 128.1, 127.9, 126.7, 126.2 (q, *J* = 3.6 Hz), 125.5, 124.2, 123.9 (q, *J* = 272.9 Hz), 119.8. Anal. Calcd. for C_21_H_12_Cl_2_F_3_NO_3_ (454.23): C, 55.53; H, 2.66; N, 3.08. Found: C, 55.69; H, 2.47; N, 2.86.

*4-Chloro-2-(3,4-dichlorophenylcarbamoyl)phenyl-4-(trifluoromethyl)benzoate* (**2e**). Yield 73%; m.p.: 156–158.5 °C. IR (ATR): 3313, 3079, 2933, 2858, 1718 (CO ester), 1671, 1650, 1627, 1580, 1518, 1478, 1451, 1384, 1323, 1278, 1249, 1204, 1174, 1134, 1107, 1087, 1066, 1016, 862, 823, 767, 696. ^1^H-NMR: δ 10.80 (1H, bs, NH), 8.25 (2H, d, *J* = 8.1 Hz, H2′′, H6′′), 7.94 (2H, d, *J* = 8.3 Hz, H3′′, H5′′), 7.90–7.87 (2H, m, H2′, H3), 7.75 (1H, dd, *J* = 2.6 Hz, *J* = 8.7 Hz, H5), 7.61 (1H, d, *J* = 8.8 Hz, H6), 7.56–7.52 (2H, m, H5′, H6′). ^13^C-NMR: δ 163.3, 162.9, 146.9, 138.9, 133.7 (q, *J* = 32.1 Hz), 132.4, 132.1, 131.1, 130.9, 130.8, 130.7, 130.6, 129.3, 126.2 (q, *J* = 3.7 Hz), 125.7, 125.6, 123.9 (q, *J* = 272.8 Hz), 121.2, 120.0. Anal. Calcd. for C_21_H_11_Cl_3_F_3_NO_3_ (488.67): C, 51.61; H, 2.27; N, 2.87. Found: C, 51.77; H, 2.41; N, 2.64.

*5-Chloro-2-(3,4-dichlorophenylcarbamoyl)phenyl-4-(trifluoromethyl)benzoate* (**2f**). Yield 74%; m.p.: 144–146 °C. IR (ATR): 3306, 29333, 2858, 1748 (CO ester), 1699, 1649, 1601, 1581, 1525, 1477, 1452, 1409, 1379, 1325, 1261, 1250, 1194, 1164, 1130, 1120, 1068, 1016, 864, 769, 698. ^1^H-NMR: δ 10.77 (1H, bs, NH), 8.25 (2H, d, *J* = 8.1 Hz, H2′′, H6′′), 7.94 (2H, d, *J* = 8.3 Hz, H3′′, H5′′), 7.91 (1H, s, H2′), 7.82 (1H, d, *J* = 8.3 Hz, H3), 7.74 (1H, d, *J* = 2.0 Hz, H6), 7.61 (1H, dd, *J* = 2.0 Hz, *J* = 8.5 Hz, H4), 7.57–7.52 (2H, m, H5′, H6′). ^13^C-NMR: δ 163.4, 163.2, 148.8, 138.9, 136.1, 133.7 (q, *J* = 31.8 Hz), 132.4, 131.1, 131.0, 130.9, 130.8, 127.9, 126.8, 126.2 (q, *J* = 3.7 Hz), 125.6, 124.0, 123.8 (q, *J* = 272.3 Hz), 121.1, 120.0. Anal. Calcd. for C_21_H_11_Cl_3_F_3_NO_3_ (488.67): C, 51.61; H, 2.27; N, 2.87. Found: C, 51.48; H, 2.11; N, 2.98.

*2-(3-Bromophenylcarbamoyl)-4-chlorophenyl-4-(trifluoromethyl)benzoate* (**2g**). Yield 49%; m.p.: 196–198 °C. IR (ATR): 3319, 2932, 2853, 1717 (CO ester), 1666, 1650, 1625, 1573, 1521, 1478, 1451, 1410, 1323, 1291, 1271, 1241, 11167, 1132, 1112, 1088, 1066, 1016, 893, 860, 766, 696, 686. ^1^H-NMR: δ 10.68 (1H, bs, NH), 8.26 (2H, d, *J* = 8.1 Hz, H2′′, H6′′), 7.95 (2H, d, *J* = 8.2 Hz, H3′′, H5′′), 7.88–7.85 (2H, m, H2′, H3), 7.75 (1H, dd, *J* = 2.6 Hz, *J* = 8.7 Hz, H5), 7.63 (1H, d, *J* = 8.0 Hz, H6), 7.54 (1H, d, *J* = 8.7 Hz, H6′), 7.26–7.23 (2H, m, H4′, H5′). ^13^C-NMR: δ 163.3, 162.8, 146.9, 140.4, 133.7 (q, *J* = 32.1 Hz), 132.4, 131.9, 130.9, 130.9, 130.8, 130.7, 129.3, 126.8, 126.2 (q, *J* = 3.7 Hz), 125.6, 123.8 (q, *J* = 268.2 Hz), 122.4, 121.6, 118.8. Anal. Calcd. for C_21_H_12_BrClF_3_NO_3_ (498.68): C, 50.58; H, 2.43; N, 2.81. Found: C, 50.72; H, 2.19; N, 2.55.

*2-(3-Bromophenylcarbamoyl)-5-chlorophenyl-4-(trifluoromethyl)benzoate* (**2h**). Yield 85%; m.p.: 166–168.5 °C. IR (ATR): 3305, 2933, 2857, 1738 (CO ester), 1650, 1589, 1537, 1479, 1452, 1411, 1323, 1258, 1242, 1169, 1130, 1111, 1068, 1016, 861, 770, 698, 675. ^1^H-NMR: δ 10.65 (1H, bs, NH), 8.26 (2H, d, *J* = 7.9 Hz, H2′′, H6′′), 7.94 (2H, d, *J* = 7.9 Hz, H3′′, H5′′), 7.85 (1H, s, H2′), 7.82 (1H, d, *J* = 8.3 Hz, H3), 7.72 (1H, d, *J* = 1.9 Hz, H6), 7.65–7.53 (2H, m, H4, H6′), 7.31 (1H, m, H5′), 7.23 (1H, m, H4′). ^13^C-NMR: δ 163.4, 163.2, 148.8, 140.4, 136.0, 133.7 (q, *J* = 32.3 Hz), 131.1, 130.9, 130.8, 128.2, 127.9, 126.7, 126.6, 126.2 (q, *J* = 3.6 Hz), 123.9, 123.4 (q, *J* = 272.9 Hz), 122.3, 121.6, 118.8. Anal. Calcd. for C_21_H_12_BrClF_3_NO_3_ (498.68): C, 50.58; H, 2.43; N, 2.81. Found: C, 50.46; H, 2.58; N, 2.71.

*2-(4-Bromophenylcarbamoyl)-4-chlorophenyl-4-(trifluoromethyl)benzoate* (**2i**). Yield 53%; m.p.: 173–175.5 °C. IR (ATR): 3329, 2932, 2857, 1717 (CO ester), 1672, 1650, 1523, 1487, 1451, 1394, 1323, 1277, 1242, 1203, 1172, 1133, 1112, 1088, 1066, 1016, 860, 817, 767, 695. ^1^H-NMR: δ 10.66 (1H, bs, NH), 8.25 (2H, d, *J* = 8.1 Hz, H2′′, H6′′), 7.94 (2H, d, *J* = 8.3 Hz, H3′′, H5′′), 7.86 (1H, d, *J* = 2.6 Hz, H6), 7.73 (1H, dd, *J* = 2.6 Hz, *J* = 8.7 Hz, H5), 7.63 (1H, d, *J* = 8.1 Hz, H3), 7.58-7.52 (2H, m, H2′, H6′), 7.47-7.44 (2H, m, H3′, H5′). ^13^C-NMR: δ 163.3, 162.7, 146.9, 138.2, 133.6 (q, *J* = 32.0 Hz), 132.5, 131.8, 131.7, 130.9, 130.8, 130.6, 129.2, 126.2 (q, *J* = 3.7 Hz), 125.6, 123.8 (q, *J* = 273.0 Hz), 121.9, 115.8. Anal. Calcd. for C_21_H_12_BrClF_3_NO_3_ (498.68): C, 50.58; H, 2.43; N, 2.81. Found: C, 50.40; H, 2.67; N, 2.64.

*2-(4-Bromophenylcarbamoyl)-5-chlorophenyl-4-(trifluoromethyl)benzoate* (**2j**). Yield 72%; m.p.: 152.5–154.5 °C. IR (ATR): 3291, 2933, 1737 (CO ester), 1650, 1602, 1544, 1489, 1449, 1406, 1325, 1257, 1241, 1182, 1156, 1128, 1111, 1069, 1015, 893, 861, 824, 770, 698, 667. ^1^H-NMR: δ 10.63 (1H, bs, NH), 8.25 (2H, d, *J* = 8.1 Hz, H2′′, H6′′), 7.94 (2H, d, *J* = 8.3 Hz, H3′′, H5′′), 7.81 (1H, d, *J* = 8.3 Hz, H3), 7.71 (1H, d, *J* = 2.1 Hz, H6), 7.64–7.55 (3H, m, H4, H2′, H6′), 7.46–7.43 (2H, m, H3′, H5′). ^13^C-NMR: δ 163.2, 162.7, 148.8, 138.3, 135.9, 133.7 (q, *J* = 32.1 Hz), 132.4, 131.7, 131.0, 130.9, 128.3, 126.7, 126.2 (q, *J* = 3.7 Hz), 123.9, 123.8 (q, *J* = 272.8 Hz), 121.9, 115.7. Anal. Calcd. for C_21_H_12_BrClF_3_NO_3_ (498.68): C, 50.58; H, 2.43; N, 2.81. Found: C, 50.36; H, 2.65; N, 2.94.

*4-Chloro-2-(3-fluorophenylcarbamoyl)phenyl-4-(trifluoromethyl)benzoate* (**2k**). Yield 76%; m.p.: 151–153.5 °C. IR (ATR): 3317, 2933, 2857, 1721 (CO ester), 1699, 1650, 1543, 1444, 1410, 1323, 1272, 1236, 1167, 1154, 1130, 1115, 1089, 1065, 1020, 859, 844, 766, 686. ^1^H-NMR: δ 10.73 (1H, bs, NH), 8.26 (2H, d, *J* = 7.9 Hz, H2′′, H6′′), 7.94 (2H, d, *J* = 7.9 Hz, H3′′, H5′′), 7.86 (1H, s, H3), 7.73 (1H, d, *J* = 8.5 Hz, H5), 7.64–7.49 (2H, m, H6, H6′), 7.39–7.29 (2H, m, H2′, H5′), 6.88 (1H, t, *J* = 8.3 Hz, H4′). ^13^C-NMR: δ 163.3, 162.8, 162.1 (d, *J* = 241.5 Hz), 146.9, 140.5 (d, *J* = 11.1 Hz), 133.7 (q, *J* = 31.7 Hz), 132.5, 131.9, 130.9, 130.7, 130.5 (d, *J* = 9.4 Hz), 129.2, 127.9, 126.2 (q, *J* = 3.6 Hz), 125.6, 123.8 (q, *J* = 273.0 Hz), 115.8 (d, *J* = 2.8 Hz), 110.6 (d, *J* = 21.2 Hz), 106.7 (d, *J* = 26.2 Hz). Anal. Calcd. for C_21_H_12_ClF_4_NO_3_ (437.77): C, 57.62; H, 2.76; N, 3.20. Found: C, 57.88; H, 2.99; N, 3.03.

*5-Chloro-2-(3-fluorophenylcarbamoyl)phenyl-4-(trifluoromethyl)benzoate* (**2l**). Yield 55%; m.p.: 169–171.5 °C. IR (ATR): 3320, 2932, 2852, 1739 (CO ester), 1651, 1624, 1602, 1569, 1548, 1491, 1414, 1325, 1259, 1242, 1200, 1172, 1138, 1122, 1070, 1016, 861, 770, 700, 669. ^1^H-NMR: δ 10.70 (1H, bs, NH), 8.26 (2H, d, *J* = 8.0 Hz, H2′′, H6′′), 7.94 (2H, d, *J* = 8.1 Hz, H3′′, H5′′), 7.82 (1H, d, *J* = 8.3 Hz, H3), 7.72 (1H, d, *J* = 1.8 Hz, H6), 7.59 (1H, dd, *J* = 1.7 Hz, *J* = 8.3 Hz, H4), 7.52 (1H, d, *J* = 11.4 Hz, H6′), 7.38–7.28 (2H, m, H2′, H5′), 6.87 (1H, t, *J* = 8.4 Hz, H4′). ^13^C-NMR: δ 163.4, 163.2, 162.1 (d, *J* = 241.5 Hz), 148.8, 140.6 (d, *J* = 10.9 Hz), 135.9, 133.7 (q, *J* = 32.1 Hz), 132.4, 131.0, 130.9, 130.5 (d, *J* = 9.4 Hz), 128.3, 126.7, 126.2 (q, *J* = 3.7 Hz), 124.0, 123.8 (q, *J* = 272.8 Hz), 115.7 (d, *J* = 2.8 Hz), 110.5 (d, *J* = 21.0 Hz), 106.7 (d, *J* = 26.2 Hz). Anal. Calcd. for C_21_H_12_ClF_4_NO_3_ (437.77): C, 57.62; H, 2.76; N, 3.20. Found: C, 57.39; H, 3.02; N, 3.47.

*4-Chloro-2-(4-fluorophenylcarbamoyl)phenyl-4-(trifluoromethyl)benzoate* (**2m**). Yield 70%; m.p.: 181–183.5 °C. IR (ATR): 3329, 2933, 2858, 1721 (CO ester), 1665, 1650, 1572, 1530, 1508, 1479, 1451, 1408, 1323, 1279, 1235, 1167, 1132, 1114, 1087, 1065, 1016, 825, 767, 695. ^1^H-NMR: δ 10.58 (1H, bs, NH), 8.25 (2H, d, *J* = 7.8 Hz, H2′′, H6′′), 7.94 (2H, d, *J* = 7.9 Hz, H3′′, H5′′), 7.85 (1H, d, *J* = 2.4 Hz, H3), 7.77–7.73 (1H, dd, *J* = 2.4 Hz, *J* = 8.7 Hz, H5), 7.64–7.51 (3H, m, H6, H2′, H6′), 7.13–7.09 (2H, m, H3′, H5′). ^13^C-NMR: δ 163.3, 162.5, 158.5 (d, *J* = 240.6 Hz), 146.9, 135.2 (d, *J* = 2.6 Hz), 133.6 (q, *J* = 32.0 Hz), 132.5, 131.7, 131.0, 130.9, 130.6, 129.2, 126.2 (q, *J* = 3.8 Hz), 125.6, 123.8 (q, *J* = 272.9 Hz), 121.9 (d, *J* = 7.9 Hz), 115.5 (d, *J* = 22.3 Hz). Anal. Calcd. for C_21_H_12_ClF_4_NO_3_ (437.77): C, 57.62; H, 2.76; N, 3.20. Found: C, 57.95; H, 2.52; N, 3.26.

*5-Chloro-2-(4-fluorophenylcarbamoyl)phenyl-4-(trifluoromethyl)benzoate* (**2n**). Yield 72%; m.p.: 176.5–179 °C. IR (ATR): 3294, 2933, 2857, 1737 (CO ester), 1646, 1602, 1556, 1508, 1452, 1413, 1328, 1260, 1234, 1192, 1156, 1137, 1126, 1111, 1069, 1016, 861, 833, 771, 699, 669. ^1^H-NMR: δ 10.55 (1H, bs, NH), 8.26 (2H, d, *J* = 7.8 Hz, H2′′, H6′′), 7.94 (2H, d, *J* = 7.8 Hz, H3′′, H5′′), 7.81 (1H, d, *J* = 8.2 Hz, H3), 7.71 (1H, d, *J* = 1.9 Hz, H6), 7.64–7.59 (3H, m, H4, H2′, H6′), 7.13–7.08 (2H, m, H3′, H5′). ^13^C-NMR: δ 163.2, 163.0, 158.5 (d, *J* = 240.5 Hz), 148.8, 135.7, 135.2 (d, *J* = 2.5 Hz), 133.7 (q, *J* = 32.2 Hz), 132.5, 131.0, 130.9, 128.5, 126.7, 126.2 (q, *J* = 3.7 Hz), 123.9, 123.8 (q, *J* = 272.9 Hz), 121.8 (d, *J* = 7.9 Hz), 115.5 (d, *J* = 22.2 Hz). Anal. Calcd. for C_21_H_12_ClF_4_NO_3_ (437.77): C, 57.62; H, 2.76; N, 3.20. Found: C, 57.94; H, 2.59; N, 2.94.

*4-Chloro-2-(3-(trifluoromethyl)phenylcarbamoyl)phenyl-4-(trifluoromethyl)benzoate* (**2o**). Yield 68%; m.p.: 158.5–160.5 °C. IR (ATR): 3306, 2933, 2858, 1719 (CO ester), 1699, 1670, 1650, 1543, 1450, 1409, 1323, 1276, 1236, 1168, 1131, 1108, 1066, 1020, 844, 766, 696, 687. ^1^H-NMR: δ 10.84 (1H, bs, NH), 8.26 (2H, d, *J* = 8.1 Hz, H2′′, H6′′), 7.94–7.84 (4H, m, H3, H2′, H3′′, H5′′), 7.76 (1H, dd, *J* = 8.7 Hz, *J* = 2.5 Hz, H5), 7.63 (1H, d, *J* = 8.0 Hz, H6′), 7.56 (1H, d, *J* = 8.7 Hz, H6), 7.52 (1H, t, *J* = 7.9 Hz, H5′), 7.40 (1H, d, *J* = 7.8 Hz, H4′). ^13^C-NMR: δ 163.3, 163.0, 146.9, 139.5, 133.7 (q, *J* = 32.1 Hz), 132.4, 132.0, 130.9, 130.8, 130.7, 130.2, 129.8 (q, *J* = 29.8 Hz), 129.3, 126.1 (q, *J* = 3.7 Hz), 125.5, 124.4 (q, *J* = 263.2 Hz), 123.8 (q, *J* = 272.2 Hz), 123.6, 120.5 (q, *J* = 3.7 Hz), 116.2 (q, *J* = 3.7 Hz). Anal. Calcd. for C_22_H_12_ClF_6_NO_3_ (487.78): C, 54.17; H, 2.48; N, 2.87. Found: C, 54.02; H, 2.57; N, 2.99.

*5-Chloro-2-(3-(trifluoromethyl)phenylcarbamoyl)phenyl-4-(trifluoromethyl)benzoate* (**2p**). Yield 50%; m.p.: 160–162.5 °C. IR (ATR): 3290, 2934, 2858, 1739 (CO ester), 1699, 1650, 1601, 1558, 1543, 1492, 1413, 1322, 1259, 1246, 1164, 1133, 1067, 1016, 884, 862, 802, 771, 697. ^1^H-NMR: δ 10.80 (1H, bs, NH), 8.27 (2H, d, *J* = 8.1 Hz, H2′′, H6′′), 7.95–7.83 (5H, m, H3, H2′, H6′, H3′′, H5′′), 7.73 (1H, d, *J* = 2.1 Hz, H6), 7.60 (1H, dd, *J* = 8.3 Hz, *J* = 2.0 Hz, H4), 7.51 (1H, t, *J* = 8.0 Hz, H5′), 7.38 (1H, d, *J* = 7.8 Hz, H4′). ^13^C-NMR: δ 163.5, 163.2, 148.8, 139.6, 136.1, 133.7 (q, *J* = 32.1 Hz), 132.3, 131.1, 130.9, 130.1, 129.8 (q, *J* = 30.7 Hz), 128.2, 126.8, 126.1 (q, *J* = 3.7 Hz), 124.1 (q, *J* = 272.3 Hz), 123.9, 123.8 (q, *J* = 272.9 Hz), 123.5, 120.4 (q, *J* = 3.9 Hz), 116.1 (q, *J* = 4.1 Hz). Anal. Calcd. for C_22_H_12_ClF_6_NO_3_ (487.78): C, 54.17; H, 2.48; N, 2.87. Found: C, 54.46; H, 2.65; N, 2.67.

*4-Chloro-2-(4-(trifluoromethyl)phenylcarbamoyl)phenyl-4-(trifluoromethyl)benzoate* (**2q**). Yield 75%; m.p.: 166.5–169 °C. IR (ATR): 3330, 2933, 2857, 1717 (CO ester), 1676, 1650, 1527, 1478, 1452, 1409, 1322, 1280, 1257, 1237, 1168, 1126, 1110, 1088, 1066, 1017, 861, 843, 767, 697. ^1^H-NMR: δ 10.89 (1H, bs, NH), 8.25 (2H, d, *J* = 8.1 Hz, H2′′, H6′′), 7.92 (2H, d, *J* = 8.2 Hz, H3′′, H5′′), 7.90 (1H, d, *J* = 2.6 Hz, H3), 7.81 (2H, d, *J* = 8.5 Hz, H2′, H6′), 7.76 (1H, dd, *J* = 2.6 Hz, *J* = 8.7 Hz, H5), 7.64 (2H, d, *J* = 8.7 Hz, H3′, H5′), 7.55 (1H, d, *J* = 8.7 Hz, H6). ^13^C-NMR: δ 163.3, 163.1, 147.0, 142.4, 133.7 (q, *J* = 32.1 Hz), 132.4, 132.0, 130.9, 130.8, 130.7, 129.3, 126.2 (m), 125.6, 124.5 (q, *J* = 271.4 Hz), 124.4 (q, *J* = 31.8 Hz), 123.9 (q, *J* = 272.9 Hz), 119.9. Anal. Calcd. For C_22_H_12_ClF_6_NO_3_ (487.78): C, 54.17; H, 2.48; N, 2.87. Found: C, 54.42; H, 2.71; N, 3.14.

*5-Chloro-2-(4-(trifluoromethyl)phenylcarbamoyl)phenyl-4-(trifluoromethyl)benzoate* (**2r**). Yield 70%; m.p.: 142–143.5 °C. IR (ATR): 3346, 2932, 2856, 1719 (CO ester), 1671, 1649, 1602, 1524, 1485, 1451, 1407, 1321, 1281, 1251, 1179, 1165, 1120, 1089, 1064, 1017, 863, 843, 824, 761, 697. ^1^H-NMR: δ 10.85 (1H, bs, NH), 8.25 (2H, d, *J* = 8.1 Hz, H2′′, H6′′), 7.93 (2H, d, *J* = 8.2 Hz, H3′′, H5′′), 7.85 (1H, d, *J* = 8.3 Hz, H3), 7.80 (2H, d, *J* = 8.3 Hz, H2′, H6′), 7.73 (1H, d, *J* = 2.0 Hz, H6), 7.63 (2H, d, *J* = 8.6 Hz, H3′, H5′), 7.60 (1H, dd, *J* = 2.1 Hz, *J* = 8.3 Hz, H4). ^13^C-NMR: δ 163.6, 163.2, 148.9, 142.5, 136.1, 133.7 (q, *J* = 32.1 Hz), 132.4, 131.1, 130.9, 128.1, 127.8, 126.7, 126.1 (m), 124.4 (q, *J* = 271.3 Hz), 124.0 (q, *J* = 32.0 Hz), 123.8 (q, *J* = 273.0 Hz), 119.8. Anal. Calcd. for C_22_H_12_ClF_6_NO_3_ (487.78): C, 54.17; H, 2.48; N, 2.87. Found: C, 54.33; H, 2.65; N, 2.69.

*4-Bromo-2-(4-(trifluoromethyl)phenylcarbamoyl)phenyl-4-(trifluoromethyl)benzoate* (**2s**). Yield 67%; m.p.: 162–163.5 °C. IR (ATR): 3325, 2933, 2858, 1717 (CO ester), 1699, 1674, 1650, 1528, 1452, 1408, 1322, 1279, 1236, 1167, 1129, 1110, 1089, 1066, 1017, 844, 766, 687. ^1^H-NMR: δ 10.88 (1H, bs, NH), 8.25 (2H, d, *J* = 8.1 Hz, H2′′, H6′′), 8.03 (1H, d, *J* = 2.4 Hz, H3), 7.92 (2H, d, *J* = 8.3 Hz, H3′′, H5′′), 7.88 (1H, dd, *J* = 2.6 Hz, *J* = 8.8 Hz, H5), 7.80 (2H, d, *J* = 8.5 Hz, H2′, H6′), 7.66–7.61 (2H, m, H3′, H5′), 7.48 (1H, d, *J* = 8.7 Hz, H6). ^13^C-NMR: δ 163.3, 163.0, 147.4, 142.4, 135.0, 133.7 (q, *J* = 32.3 Hz), 132.4, 132.0, 131.0, 130.9, 126.2 (m), 125.9, 124.4 (q, *J* = 32.0 Hz), 124.8 (q, *J* = 274.1 Hz), 123.5 (q, *J* = 268.6 Hz), 119.9, 118.8. Anal. Calcd. for C_22_H_12_BrF_6_NO_3_ (532.23): C, 49.65; H, 2.27; N, 2.63. Found: C, 49.43; H, 2.12; N, 2.54.

### 3.4. Antifungal Activity Determination

The antifungal properties of all synthesized compounds were evaluated *in vitro* against four *Candida* strains (*Candida albicans* ATCC 44859, *Candida tropicalis* 156, *Candida krusei* E28, and *Candida glabrata* 20/I), *Trichosporon asahii* 1188 and three filamentous fungi (*Aspergillus fumigatus* 231, *Absidia corymbifera* 272, and *Trichophyton mentagrophytes* 445).

The microdilution broth method was used according to the CLSI M27-A3 and M38-A2 guidelines for yeasts [26] and moulds [27] in RPMI 1640 with glutamine (KlinLab, Prague, Czech Republic) buffered to pH 7.0 with 0.165 mol of 3-morpholino-propane-1-sulphonic acid (Sigma-Aldrich, Seelze, Germany). DMSO served as a diluent for all compounds. In the yeast, the final size of the inoculum was 5 × 10^3^ ± 0.2 CFU/mL. For the moulds “*Aspergillus*, *Trichophyton* and *Absidia corymbifera*”, the spores were harvested after the cultivation of a given fungal strain grown on Sabouraud agar from 3 to 10 days. The final size of the inoculum was 0.5–5 ×10^4^ CFU/mL. The sizes of the fungal inocula were checked using a Bürker’s chamber. Fluconazole was used as a reference specific drug and undecylenic acid as a non-specific one. The minimum inhibitory concentrations (MIC) were assayed as an 80% (IC_80_) or higher reduction of growth in comparison to the control (for salicylanilides and for yeasts in the case of 4-(trifluoromethyl)benzoates) or as an 50% (IC_50_) or higher reduction of growth (for the activity of 4-(trifluoromethyl)benzoates towards filamentous fungi) in comparison to the control. The determination of results was performed visually and spectrophotometrically (at 540 nm). The MIC values were determined after 24 and 48 h of incubation in the dark at 35 °C (±0.1) in a humid atmosphere; only for *T. mentagrophytes* the final MIC were determined after 72 and 120 h of incubation. MICs were determined twice and in duplicate.

## 4. Conclusions

In summary, we have synthesized nineteen salicylanilides and eighteen corresponding esters with 4-(trifluoromethyl)benzoic acid. New compounds were characterised and all derivatives were evaluated as potential antimycotic agents towards eight fungal strains. It was not possible to determine MICs of a range of the esters because of a low solubility and/or a precipitation in the testing medium. Salicylanilides and their esters affected the fungal growth from 0.49 µmol/L; however, their activity is not uniform and especially against *Candida* spp. it seems to be most likely sporadic. However, some of the here described compounds may represent derivatives with a promising *in vitro* antifungal properties.
